# The Roles of Tricellular Tight Junction Protein Angulin-1/Lipolysis-Stimulated Lipoprotein Receptor (LSR) in Endometriosis and Endometrioid-Endometrial Carcinoma

**DOI:** 10.3390/cancers13246341

**Published:** 2021-12-17

**Authors:** Hiroshi Shimada, Takayuki Kohno, Takumi Konno, Tadahi Okada, Kimihito Saito, Yuma Shindo, Shin Kikuchi, Mitsuhiro Tsujiwaki, Marie Ogawa, Motoki Matsuura, Tsuyoshi Saito, Takashi Kojima

**Affiliations:** 1 Department of Cell Science, Research Institute for Frontier Medicine, Sapporo Medical University School of Medicine, Sapporo 060-8556, Japan; hshimada1984@cap.ocn.ne.jp (H.S.); t.konno1225@gmail.com (T.K.); tadahi.okada@gmail.com (T.O.); kimihito.saito@sapmed.ac.jp (K.S.); shindo.y@sapmed.ac.jp (Y.S.); 2 Departments of Obstetrics, Sapporo Medical University School of Medicine, Sapporo 060-8556, Japan; marie1221inouse@yahoo.co.jp (M.O.); motoki.gyne@gmail.com (M.M.); tsaito@sapmed.ac.jp (T.S.); 3 Department of Anatomy, Sapporo Medical University School of Medicine, Sapporo 060-8556, Japan; ksin@sapmed.ac.jp; 4 Department of Pathology, Sapporo Medical University School of Medicine, Sapporo 060-8556, Japan; auditt3200@gmail.com

**Keywords:** endometrioid-endometrial carcinoma, endometriosis, tricellular tight junctions, angulin-1/LSR, ASPP2, claudins, EMT, Hippo pathway, HDAC, AMPK

## Abstract

**Simple Summary:**

Abnormality of tight junction proteins closely contributes to epithelial–mesenchymal transition (EMT) and the malignancy of various cancers. Angulin-1/lipolysis-stimulated lipoprotein receptor (LSR) is a novel molecular constituent of tricellular contacts that has a barrier function. Loss of angulin-1/LSR correlates with the malignancy in various cancers, including endometrioid-endometrial carcinoma (EEC). Moreover, loss of angulin-1/LSR upregulates claudin-1, and loss of apoptosis-stimulating p53 protein 2 (ASPP2) at tricellular contacts downregulates angulin-1/LSR in human EEC cell line Sawano. Angulin-1/LSR and ASPP2 concentrate at both midbody and centrosome during cytokinesis in Sawano. In EEC tissues, angulin-1/LSR and ASPP2 are reduced and claudin-2 is overexpressed during malignancy, while in the tissues of endometriosis changes in localization of angulin-1/LSR and claudin-2 are seen. This review highlights how the loss of angulin-1/LSR promotes the progression of endometriosis and EEC and discusses the possibility of therapeutic targeting for angulin-1/LSR via multiple signaling pathways and its related proteins.

**Abstract:**

Tight junction proteins play roles beyond permeability barriers functions and control cell proliferation and differentiation. The relation between tight junctions and the signal transduction pathways affects cell growth, invasion and migration. Abnormality of tight junction proteins closely contributes to epithelial mesenchymal transition (EMT) and malignancy of various cancers. Angulin-1/lipolysis-stimulated lipoprotein receptor (LSR) forms tricellular contacts that has a barrier function. Downregulation of angulin-1/LSR correlates with the malignancy in various cancers, including endometrioid-endometrial carcinoma (EEC). These alterations have been shown to link to not only multiple signaling pathways such as Hippo/YAP, HDAC, AMPK, but also cell metabolism in ECC cell line Sawano. Moreover, loss of angulin-1/LSR upregulates claudin-1, and loss of apoptosis stimulating p53 protein 2 (ASPP2) downregulates angulin-1/LSR. Angulin-1/LSR and ASPP2 concentrate at both midbody and centrosome in cytokinesis. In EEC tissues, angulin-1/LSR and ASPP2 are reduced and claudin-2 is overexpressed during malignancy, while in the tissues of endometriosis changes in localization of angulin-1/LSR and claudin-2 are seen. This review highlights how downregulation of angulin-1/LSR promotes development of endometriosis and EEC and discusses about the roles of angulin-1/LSR and its related proteins, including claudins and ASPP2.

## 1. Introduction

Endometrial cancer (EC) is the most frequent neoplasia of the female genital tract in the world in 2018 [1], and its incidence in industrialized countries, as well as Japan, is recently increasing [1,2]. The prevalence of EC in young obese women under the age of 50 years has been increasing accordingly to the data from the Surveillance, Epidemiology, and End Results program from 1990 to the present [3]. While the overall survival rate of patients with early stage (FIGO Stage I and II) EC is high, that of patients with advanced stage (FIGO Stages III and IV) or recurrent EC is poor (Japan Society of Obstetrics and Gynecology. Patient Annual Report for 2016). Actually, the treatment options for advanced or recurrent EC have not improved [4]. Thus, further research on the pathophysiology of endometrial cancer must be conducted to develop novel therapies.

Research in the field of molecular biology has recently been developing, and molecular-targeted therapy has become a new treatment strategy for various cancers. Several studies show that changed expression of some genes such as PTEN, KRAS, CTNNB1, PIK3CA and FGFR2 is detected in EC tissues and cells [5]. Indeed, several clinical trials of molecular-targeted therapy in EC are ongoing [6]. However, in the clinical setting, little molecular-targeted medicine can be used for EC treatment. Therefore, more studies are needed to determine the appropriate molecular-targeted therapy for EC, including identification of novel signal pathways in the progression of EC cells.

The epithelial–mesenchymal transition (EMT), which is an essential step in cancer progression, can cause cancer cells to lose their cell–cell adhesions, including tight junctions, and acquire the ability to migrate and proliferate [7,8]. Several studies have reported that expression of tight junction proteins plays an important role in the EMT of cancer cells [8]. These alterations are regulated via not only multiple signaling pathways such as Hippo/YAP, HDAC, JNK, AMPK, but also cell metabolism.

Thus, we have focused on tight junction proteins in the field of the molecular biology of endometriosis and endometrioid-endometrial carcinoma (EEC) [9,10,11,12,13,14]. In this review, we provide an update on the roles of tight junction proteins in endometriosis and EEC cells and discuss the possibility of targeting tight junction proteins in therapy for endometriosis and EEC.

## 2. Tight Junction Proteins in Endometriosis and Endometrial Cancer

The endometrium is a tissue that undergoes periodic changes in which the cells proliferate and differentiate regulated mainly by hormones such as estrogen and progesterone, and various cytokines. Cell–cell junctions are critical for the development and maintenance of tissues, as they regulate the flow of molecules through paracellular and intercellular pathways, including in the endometrium [15,16,17,18]. Cell–cell junctions include adherens junctions, gap junctions, desmosomes, and tight junctions (TJs) [16,18]. TJs are also associated with various signal transduction pathways that regulate epithelial cell proliferation, gene expression, differentiation, and morphogenesis [19]. TJs are composed of bicellular TJs (bTJs) and tricellular TJs (tTJs), and they form a selective barrier [20,21].

Claudins (CLDNs) are main components of TJs [22]. Overexpression of CLDN-3 and CLDN-4 is observed in EEC and may be involved in endometrial tumorgenesis [23,24]. CLDN-6 has potential as a carcinoembryonic antigen, is highly expressed in EEC, and promotes endometrial cancer progression [25,26,27].

CLDN-2 is expressed in the tight junctions of leaky epithelia, where it forms cation-selective and water permeable paracellular channels [28]. CLDN-2 expression is modulated by a variety of conditions and its overexpression increases tumorigenesis of some types of cancer cells [29]. In EEC tissues, CLDN-2 is overexpressed together with malignancy, while in endometriosis tissues a change in the localization of CLDN-2 is observed [14].

On the other hand, loss of TJs compromises cellular polarity and stimulates dedifferentiation [30,31]. Moreover, loss of several TJ proteins enhances tumor progression [32]. Loss of claudin-4 expression occurs in dedifferentiated and undifferentiated endometrial carcinomas [33] and low expression of claudin-7 in endometrial cancer cells is indicative of a late tumor stage and low histological grade [34].

tTJs are formed at the convergence of bTJs, where three epithelial cells meet in polarized epithelia [20,35]. Tricellulin (TRIC) was the first identified molecular component of tTJs [35], and angulin-1/lipolysis-stimulated lipoprotein receptor (LSR) was a novel integral membrane protein localized at tTJs [36].

**Figure 1:**
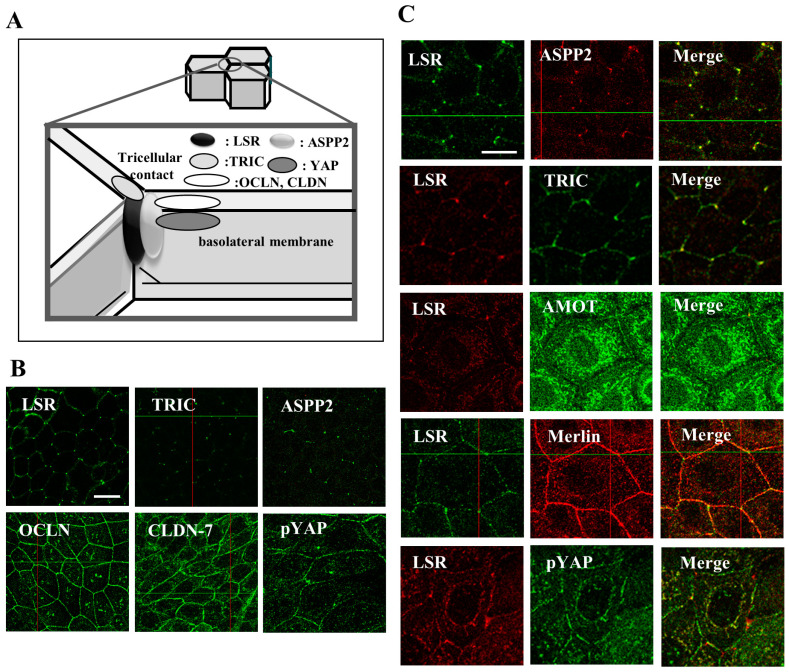
(**A**) Schematic of tight junction proteins LSR, TRIC, OCLN, CLDN, ASPP2 and pYAP at tricellular contacts in epithelial cells. (**B**) Immunocytochemial staining for LSR, TRIC, ASPP2, OCLN, CLDN-7 and pYAP. Scale bar: 20 μm. (**C**) Images of double-immunocytochemial staining for LSR, ASPP2, TRIC, AMOT, Merlin and pYAP. Scale bar: 10 μm.

Angulin-1/LSR is localized with bTJ protein CLDN-based TJ strands (Figure 1A) [36]. Angulin-1/LSR plays roles in forming the normal tTJ and the barrier function [36]. Angulin-1/LSR recruits TRIC via interaction between the cytoplasmic domain of angulin-1/LSR and the C-terminal cytoplasmic domain of TRIC [36]. More recently, it is reported that angulin-1/LSR is responsible for the plasma membrane seal at tTJ independently of TRIC and CLDNs in MDCK II cells [37].

Recently, increasing number of studies have investigated the role of angulin-1/LSR in the development of various cancers. It has been found that knockdown of angulin-1/LSR enhances cell motility and invasion in bladder cancer cells [38]. It is also reported that knockout of angulin-1/LSR in Caco-2, a colon cancer cell line, promote cell proliferation in vitro [39]. Moreover, knockdown of angulin-1/LSR promotes cell proliferation, invasion and migration in human pancreatic cancer cell lines [40]. Furthermore, downregulation of angulin-1/LSR induces malignancy via EGF-dependent CLDN-2 in a human lung adenocarcinoma cells [41]. On the other hand, the expression of angulin-1/LSR in breast cancer is higher in invasive ductal carcinomas than in invasive lobular carcinomas [42]. Furthermore, high expression of angulin-1/LSR is a poor prognostic factor in epithelial ovarian cancer and gastric cancer [43,44].

**Figure 2:**
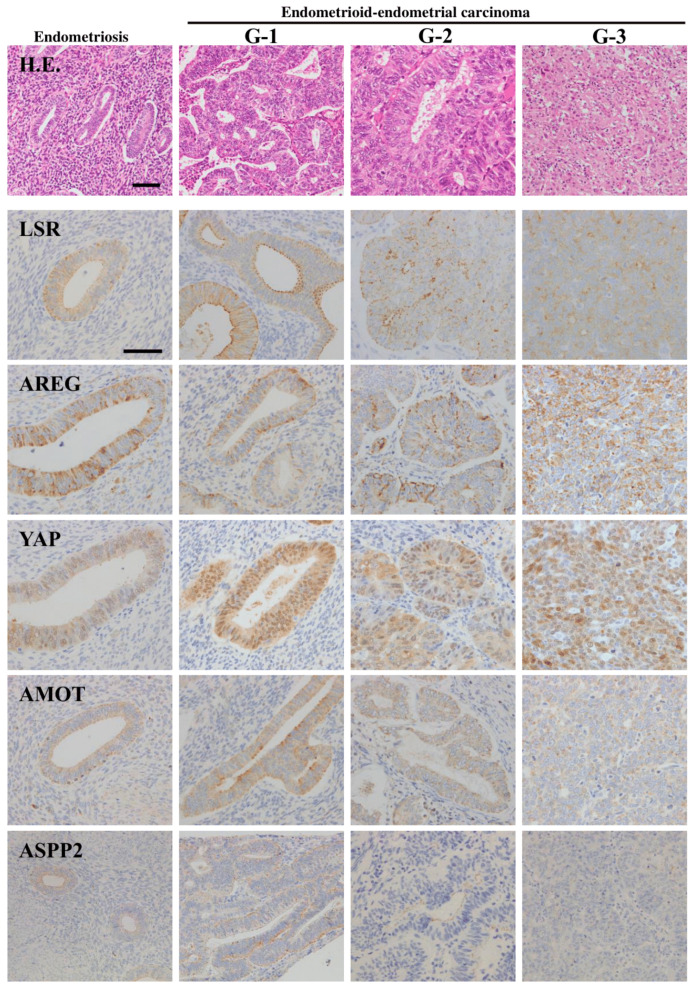
H.E. staining and immunohistochemical staining for LSR. AREG, YAP, AMOT and ASPP2 in the tissues of endometriosis and endometrioid-endometrial carcinoma (G1, G2, G3). Scale bar: 100 μm.

**Figure 3:**
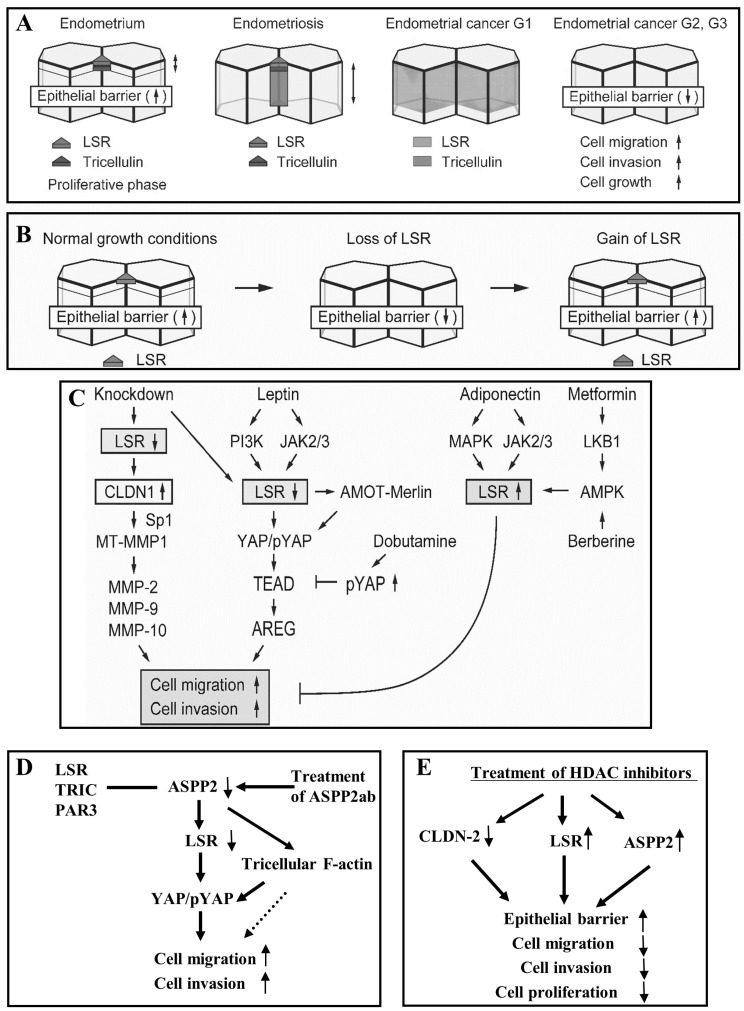
(**A**) Expression of angulin-1/LSR and tricellulin in endometriosis and endometrioid-endometrial carcinoma. (**B**) Change of epithelial barrier with change of angulin-1/LSR expression in Sawano cells. (**C**) Changes in cell functions during changes of LSR expression induced by various stimuli in Sawano cells. (**D**) Changes in cell functions induced by change of ASPP2 expression in Sawano cells. (**E**) Effects of HDAC inhibitors in Sawano cells.

In endometriosis and endometrioid-endometrial carcinoma (EEC), angulin-1/LSR is localized not only in the subapical region, but also throughout the lateral region, and angulin-1/LSR in the cancer is reduced during the malignancy [9]. Angulin-1/LSR is decreased in G2 and G3 of EEC tissues, and TRIC is reduced from G1 (Figure 2 and Figure 3A) [9]. Downregulation of angulin-1/LSR not only decreases the epithelial barrier, but also increased cell proliferation, migration and invasion of EEC cell line Sawano (Figure 3B,C) [9].

In EEC cell line Sawano, angulin-1/LSR, TRIC and ASPP2 are expressed at the tTJ and OCLN, CLDN-1, -2, -3, -4, -7, cingulin, PAR3 and YAP are observed at the bTJ (Figure 1B) [45]. Knockdown of angulin-1/LSR induces increases of CLDN-1 but not TRIC, OCLN, CLDN-3, -4, -7 in Sawano cells, whereas loss of CLDN-1 prevents the upregulation of cell invasion by loss of angulin-1/LSR [10]. The CLDN-1 promoter region contains an Sp1 binding site, and a mutation in the region results in a loss of CLDN-1 transcription [46]. Overexpression of CLDN-1 enhances cell invasion via matrix metalloproteinases (MMPs) [47,48]. Knockdown of angulin-1/LSR induces increases Sp1 and MMPs in Sawano cells [10]. These indicate that downregulation of angulin-1/LSR promotes CLDN-1 through Sp1 activity and upregulation of CLDN-1 enhances cell invasion via activation of MMPs in EEC (Figure 3C).

## 3. Hippo/YAP Pathway and Endometrial Cancer

The Hippo pathway play a crucial role in organ size and tissue homeostasis, and it is dysregulated in various cancers [49,50]. YAP and its homolog TAZ are the Hippo pathway effectors and transcriptional coactivators and form a complex with TEADs to control cell proliferation, differentiation and transformation [51]. The development of endometrial cancer contributes to the Hippo/YAP pathway [52]. YAP promotes the malignancy of endometrial cancer cells via regulation of IL-6 and IL-11 [53]. YAP/TAZ binding to the TEAD transcription factor induces amphiregulin (AREG), an epidermal growth factor receptor (EGFR) ligand [54]. The Hippo/YAP pathway plays a critical role in the pathogenesis of endometriosis [55].

Angulin-1/LSR and AREG are highly expressed in some cancer cells indicated gland-like structures in EEC tissues (Figure 2) [11]. Furthermore, angulin-1/LSR decreases and AREG increases in G2 and G3 of endometrial cancers (Figure 2). YAP expresses in the nuclei of all cells of EEC, but not endometriosis (Figure 2).

AREG and TEAD1 are markedly upregulated by downregulation of angulin-1/LSR in EEC cells, and loss of AREG prevents the cell migration and invasion induced by downregulation of angulin-1/LSR [11]. A EGF receptor inhibitor AG1478 prevents cell migration and invasion induced by AREG [11]. Loss of YAP prevents, the upregulation of AREG and TEAD1, and the cell migration and invasion induced by downregulation of LSR [11]. These findings show that the Hippo/YAP pathway is associated with cell migration and invasion in EEC cells via upregulation of TEAD1/AREG induced by loss of angulin-1/LSR.

Angiomotin (AMOT) was originally identified as an angiostatin binding protein and is associated with the pathogenesis of cancer [56]. It localizes to tight junctions and regulates the Hippo pathway (Figure 1B) [57,58]. Merlin (moesin, ezrin and radixin-like protein) encodes the *NF2* tumor suppressor gene containing an N-terminal FERM domain [59]. The recruitment of Merlin to cell junctions is crucial for a tumor suppressive function and AMOT, Merlin, Patj, and Pals1 form a tight-junction-associated protein complex [60]. Loss of AMOT/Merlin induces TEAD/AREG via the Hippo/YAP pathway and promotes the cell migration, invasion and proliferation of cancer cells [56].

AMOT is detected in G1 of EEC, whereas it is decreased in G2 and G3 (Figure 2). In Sawano cells, angulin-1/LSR is colocalized with TRIC, AMOT, Merlin and YAP at tricellular contacts (Figure 1C) [11]. Knockdown of angulin-1/LSR decreases levels of AMOT and Merlin, and increases pYAP. Knockdown of AMOT decreases Merlin and prevents the cell migration and invasion by downregulation of angulin-1/LSR (Figure 3C) [11]. Downregulation of angulin-1/LSR promotes cell invasion and migration via AMOT/Merlin in human endometrial cancer cells. These results indicate that downregulation of angulin-1/LSR may promote the malignancy via TEAD1/AREG dependent on Hippo/YAP and AMOT/Merlin in EEC.

## 4. Adipokine and Endometrial Cancer

Obesity is a risk factor for EC [61]. Women with a normal body mass index (BMI) have a 3% lifetime risk of EC, but for every 5-unit increase in the BMI, the risk of cancer increases by more than 50% [62]. Higher BMI is statistically significantly associated with poorer endometrial cancer-specific disease five-year mortality [63]. In addition, adipokines, leptin and adiponectin play important roles in the pathophysiology of cancer associated with obesity [64], although the adipokine communicates with organ systems to precisely adjust gene expression, glucoregulatory hormone exocytosis, and enzymatic action [65]. Some meta-analyses revealed that increased circulating adiponectin and adiponectin-leptin ratios and decreased leptin concentrations are associated with reduced risks of EC [66,67].

Leptin enhances cell proliferation and inhibits apoptosis of EC cells [68,69]. It also enhances human endometriotic cell migration and invasion via the JAK2/STAT3 signaling pathway [70]. Leptin signaling promotes cell invasion and the metastasis of human pancreatic cancer via JAK2/STAT3 [71]. On the other hand, adiponectin prevents leptin signaling via JAK2/STAT3 [72]. It also inhibits the progression of EC cells [73]. In EEC cells, leptin decreases angulin-1/LSR expression via PI3K and JAK2/STAT, while adiponectin increases its expression via MAPK and JAK2/STAT (Figure 3C) [9].

## 5. AMPK and Endometrial Cancer

AMP-activated protein kinase (AMPK) is known to be activated by falling cellular energy status, signaled by rising AMP/ATP and ADP/ATP ratios [74]. This energy switch controls cell growth and several other cellular processes, including lipid and glucose metabolism and autophagy [75]. AMPK activation promotes the early stages of epithelial TJ assembly [76]. AMPK regulates cell polarity and morphogenesis, as well as cell–cell junction formation through its ability to bind Par3 and the cadherin–catenin complex [75,77]. Metformin, which is a biguanide drug for type 2 DM, has been suggested to be a potential anticancer agent [78]. It directly inhibits the development of EC via the LKB1-AMPK-mTOR, PI3K-Akt, and IGF-1-associated signaling pathways, and indirectly through its effects on caspase family members and the stimulation of autophagy [79]. Moreover, metformin inhibits EMT, and many clinical trials of metformin as chemotherapy are ongoing, including large phase 3 trials [80]. The berberine also has effects on type 2 diabetes and the development of EC like those of metformin [81].

In EEC cells, metformin and berberine enhance angulin-1/LSR and inhibit the cell migration and invasion induced by downregulation of angulin-1/LSR (Figure 3C) [9]. Metaformin upregulates angulin-1/LSR via MAPK, PI3K and JAK2/STAT (Figure 3C) [9]. On the other hand, loss of angulin-1/LSR promotes cell invasion and migration via the YAP signal pathway as mentioned above [9]. As AMPK modulates Hippo/YAP pathway activity to regulate homeostasis [82], metformin might also increase expression of angulin-1/LSR and prevent induced cell migration and invasion via the Hippo/YAP pathway. Leptin decreases angulin-1/LSR expression and berberine increases its expression in EEC cells (Figure 3C) [9]. It is thought that there are anti-cancer effects of metformin and berberine via multiple mechanisms including angulin-1/LSR in endometrial cancer [83].

## 6. ASPP2 in Endometriosis and Endometrial Cancer

The apoptosis-stimulating proteins of the p53 (ASPP) family, which are identified as regulators of the tumor suppression function of p53, is compose of three members, ASPP1, ASPP2, and iASPP [84]. ASPP2 binds to various proteins that regulate apoptosis, cell polarity, proliferation, and differentiation [85,86]. p53 is a member of a family of three proteins: p53, p63, and p73. ASPP2 stimulates the apoptotic function of p53, and induces apoptosis independently of p53, which is mediated mainly by p63 and p73. ASPP2 also enhances the apoptotic functions of p63 and p73 by selectively inducing the expression of endogenous p53 target genes [87]. Loss of ASPP2 is observed during malignancy in various cancers, including EC [88,89,90,91,92].

ASPP2 regulates cell polarity and proliferation and mechanistically maintains the integrity of tight junctions and adherens junctions, including ZO-1 and β-catenin [93]. It binds PAR-3 and controls its junctional localization without affecting its expression or PAR complex binding [93]. ASPP2 plays critical roles in the establishment and maintenance of epithelial apical-basal polarity by mediating the PAR complex formation through the regulation of PAR-3 localization [94]. It is thought to be a regulator of epithelial cell polarity in cooperation with PAR-3 to form an active PAR complex [93,95]. ASPP2 also controls epithelial cell polarity via β-catenin-dependent regulation of ZEB1 [94]. ASPP2 forms an apical–lateral polarity complex at the level of tight junctions in polarized epithelial cells, acting as a scaffold for protein phosphatase 1 (PP1) and junctional YAP [96].

In endometriosis and G1 and G2 of EEC, ASPP2 expresses as well as PAR3 and angulin-1/LSR in the subapical region, whereas ASPP2 decreases in G3 of EEC (Figure 2) [13]. On the other hand, p53 expresses in most nuclei of G1 of EEC, whereas it is weakly seen in the nuclei of G3 of EEC [13].

In Sawano of EEC cells, ASPP2 is colocalized with tTJ proteins angulin-1/LSR and TRIC at tricellular contacts (Figure 1C) and binds to PAR3, angulin-1/LSR and TRIC in the confluent state, whereas ASPP2 is also colocalized with bTJ protein ZO-1 at bicellular contacts in the subconfluent state [13]. Downregulation of ASPP2 promotes cell migration and invasion with a decrease of anguin-1/LSR and an increase of phosphorylated YAP, CLDN-1, -4, and -7 (Figure 3D) [13]. Downregulation of YAP prevents phosphorylated YAP, cell migration and invasion induced by the ASPP2 suppression [13]. Treatment with a specific antibody against ASPP2 downregulates angulin-1/LSR, affects F-actin at tricellular contacts, upregulates phosphorylated YAP and CLDN-1, and induces cell migration and invasion via YAP (Figure 3D) [13]. Thus, the downregulation of ASPP2 may promote cell migration and invasion via angulin-1/LSR and the Hippo pathway.

In normal human endometrial epithelial cells, ASPP2 is colocalized with angulin-1/LSR at tricellular contacts and downregulation of ASPP2 induces CLDN-1 and -4 as well as that of angulin-1/LSR [13]. However, the detailed roles of ASPP2 remain unknown in the normal endometrial epithelial cells.

## 7. HDAC and Endometrial Cancer

Histone acetylation serves to target reader proteins and their associated complexes that carry out a wide variety of cellular functions [97]. Histone acetylation is regulated by histone acetyltransferases (HAT) and histone deacetylases (HDAC) [97,98]. According to recent studies, overexpression of HDACs is observed in various cancers [98]. HDAC inhibitors induce cancer cell cycle arrest, differentiation and cell death, reduce angiogenesis and modulate the immune response [98]. HDAC inhibitors may be useful as therapeutic drugs for treatment of EC [99].

HDAC inhibitors trichostatin A (TSA) and an inhibitor of HDAC1 and HDAC6, prevent cell proliferation, migration and invasion of human head and neck squamous cell carcinoma (HNSCC) by downregulation of p63-mediated tight junction molecules JAM-A and CLDN-1, and induction of p63 or p21-mediated growth arrest [100]. The HDAC inibitors trichostatin A (TSA) and Quisinostat (JNJ-2648158) increase angulin-1/LSR, decrease CLDN-2, promote G1 arrest and prevented the migration of lung adenocarcinoma A549 cells [101].

In Sawano of EEC cells, the HDAC inhibitor TSA, which has antitumor effects, downregulates CLDN-2, cell proliferation, invasion, and migration, and upregulates the epithelial barrier (Figure 3E) [14]. Expression of angulin-1/LSR and ASPP2 is increased by the inhibitor of HDAC1 and HDAC6, TSA (Figure 3E) [13]. The upregulation or maintenance of angulin-1/LSR and ASPP2 induced by HDAC inhibitors may be important in inhibition of the malignancy of EEC cells.

## 8. Localization of Tight Junction Proteins, YAP and ASPP2 at the Midbody and Centrosome during Cytokinesis in Endometrial Cancer Cell Line Sawano

Epithelial integrity and barrier function are maintained during cytokinesis [102,103,104,105]. The midbody is the final cellular link between the two daughter cells destined to be separated. Adherens junction and bTJ molecules concentrate in the midbody during cell division [106,107]. The bTJ molecule occludin is localized at centrosomes [108]. Furthermore, de novo tTJs present at the flank of the midbody during cytokinesis [109]. YAP also concentrated in the midbody to help cytokinesis [110].

The dynein adaptor Hook2 functions as a linker protein that binds to microtubules and organelles [111]. Hook2 is localized in the centrosome to play centrosomal function and aggresome formation, and regulate mitotic progression and cytokinesis [112,113,114]. It also interacts with the epithelial cell polarity molecules PAR3 and PAR6α and controls centrosome orientation during polarized cell migration [115].

**Figure 4:**
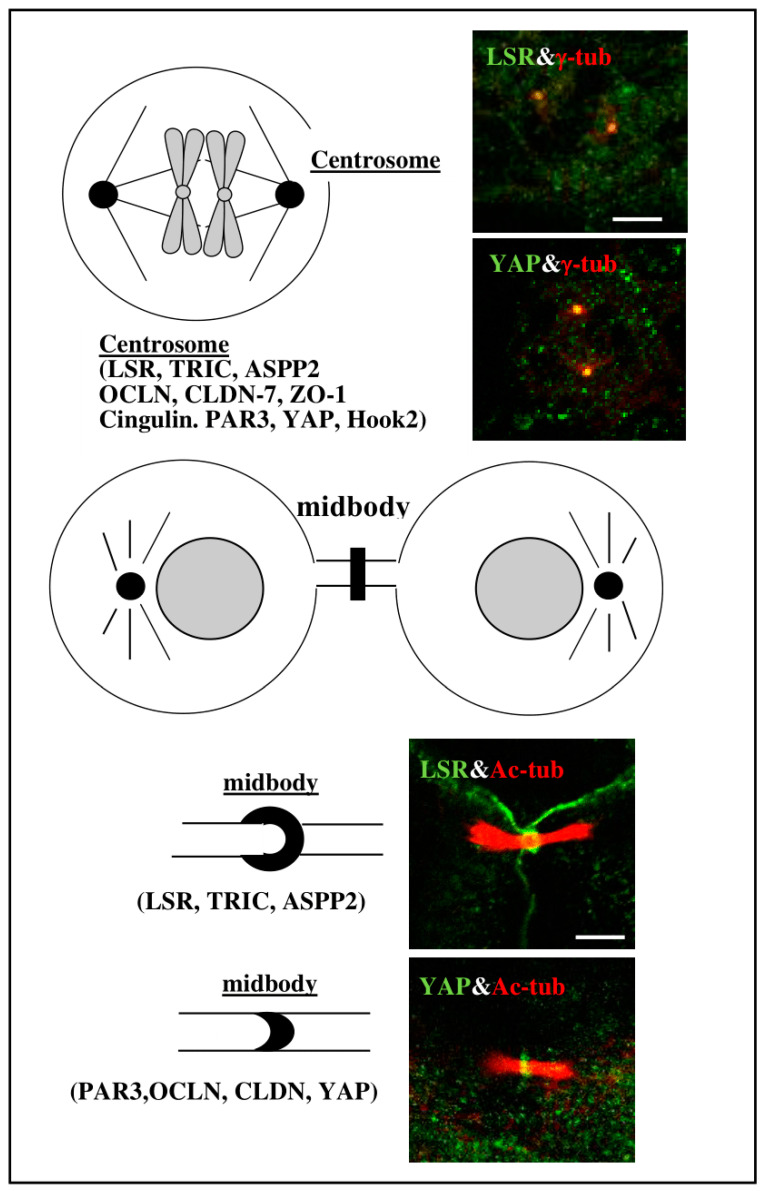
Double-immunocytochemical staining for angulin-1/LSR (green), YAP (green) and γ-tubulin (red) at centrosome in Sawano cells. Scale bar: 5 μm. Double-immunocytochemical staining for angulin-1/LSR (green), YAP (green) and acetylated-tubulin (red) at midbody in Sawano cells. Scale bar: 5 μm.

The tTJ molecules angulin-1/LSR, TRIC, ASPP2, bTJ molecules OCLN, CLDN-7, ZO-1 and CGN, the epithelial polarized related molecule PAR3 and YAP are concentrated at the flank of the acetylated tubulin-positive midbody and in γ-tubulin-positive centrosomes with the dynein adaptor Hook2 during cytokinesis in Sawano of EEC cells (Figure 4) [45]. The tTJ proteins including angulin-1/LSR, may play crucial roles not only for barrier function, but also for cytokinesis.

## 9. Conclusions

In EEC tissues, angulin-1/LSR and ASPP2 are reduced and CLDN-2 is overexpressed together with malignancy, while in endometriosis tissues a change in the localization of angulin-1/LSR and CLDN-2 is observed. Loss of angulin-1/LSR promotes the progression of endometriosis and EEC via multiple signaling pathways, Hippo/YAP, JNK, AMPK and HDAC, and CLDNs. Loss of ASPP2 at tricellular contacts in part promotes malignancy of EEC via angulin-1/LSR and YAP. The inhibitors of HDAC and signal transduction pathways may be important in therapy for endometriosis and EEC. Angulin-1/LSR also contributes to cytokinesis and cell metabolism. It is possible that angulin-1/LSR may have multiple functions in normal, endometriosis and EEC tissues.

As CLDNs are frequently dysregulated in various cancers, it is thought that they are promising biomarkers for diagnosis or targets for treatment. CLDN binders such as *Clostridium perfringens* enterotoxin and monoclonal antibodies have been tested in preclinical experiments, and some of them have progressed into clinical trials involving patients with certain cancers [116]. The angulin-1/LSR antibody can inhibit ovarian epithelial tumor growth [49]. It is possible that the antibody against N-terminal of angulin-1/LSR may be useful for therapy of endometriosis and endometrial cancer. Accordingly, angulin-1/LSR, ASPP2 and CLDN-2 may be as biomarkers for diagnosis or targets for treatment of endometriosis and EEC.
